# Portal vein stenting for variceal bleeding at the choledochojejunostomy site in a patient with portal vein occlusion or stenosis: Report of two cases

**DOI:** 10.1016/j.ijscr.2025.112068

**Published:** 2025-10-24

**Authors:** Norifumi Iseda, Ryosuke Minagawa, Koichi Kimura, Terutoshi Yamaoka, Hironori Ochi, Takashi Nishizaki

**Affiliations:** a Department of Surgery, Matsuyama Red Cross Hospital, Matsuyama, 1 Bunkyomachi, Matsuyama, Ehime, 790-8524, Japan; b Department of Vascular Surgery, Matsuyama Red Cross Hospital, Matsuyama, 1 Bunkyomachi, Matsuyama, Ehime, 790-8524, Japan; c Center for Liver-Biliary-Pancreatic Disease, Matsuyama Red Cross Hospital, Matsuyama, 1 Bunkyomachi, Matsuyama, Ehime, 790-8524, Japan

**Keywords:** Portal vein stenting, Ectopic variceal bleeding, pancreatoduodenectomy, Hepato-biliary-pancreatic surgery, Portal vein occlusion, Case report

## Abstract

**Introduction:**

Gastrointestinal bleeding following hepato-biliary-pancreatic surgery may result from variceal hemorrhage at the choledochojejunostomy site due to portal vein (PV) stenosis. However, accurately determining the underlying cause can be difficult. Moreover, managing such bleeding can be challenging and even unsuccessful.

**Presentation of case:**

Case 1: A 60-year-old man underwent pancreatoduodenectomy with PV resection and reconstruction for stage IB pancreatic cancer. Thirty-three months later, he developed variceal bleeding due to portal vein (PV) hypertension and bleeding from varices at the choledochojejunostomy site. PV stenting was performed using percutaneous transhepatic and trans-ileal approaches. No rebleeding occurred at 8 months.

Case 2: An 82-year-old man with recurrent hilar cholangiocarcinoma and PV stenosis developed variceal bleeding due to PV hypertension and variceal bleeding at the choledochojejunostomy site. After failed endoscopic therapy, PV stenting via the ileocolic vein was performed. He remained free from bleeding at 12 months.

**Discussion:**

PV stenting can be effective for controlling bleeding from choledochojejunostomy varices caused by PV stenosis or occlusion. A bidirectional approach was essential in the occluded case. Literature review indicates high success rates. The need for antithrombotic therapy post-stenting remains unclear.

**Conclusion:**

PV stenting is a feasible and effective option for variceal bleeding related to PV stenosis after pancreatoduodenectomy.

## Highlights

• Portal vein stenosis can cause variceal bleeding at choledochojejunostomy sites.
• PV stenting is a feasible option even in complete PV occlusion.
• Bidirectional approach facilitates safer guidewire passage.
• PV stenting for variceal bleeding demonstrates favorable outcomes.
• Role of antithrombotic therapy post-stenting remains uncertain.

## 1 Introduction

Gastrointestinal bleeding may arise following hepato-biliary-pancreatic surgery, and its etiology can remain unclear despite thorough diagnostic evaluation. Although portal vein (PV) stent placement is an option for treating PV obstruction, evidence supporting its efficacy in resolving bleeding from varices at a choledochojejunostomy site remains limited. The procedure itself can be technically challenging in such cases, as guidewire manipulation must often be performed without direct visualization because the PV has been occluded.

The choledochojejunostomy is created by anastomosing the common bile duct to the jejunum, and it lies in close proximity to the PV at the hepatic hilum. After pancreatic or biliary surgery, the PV runs posterior to this anastomotic site, and stenosis or occlusion of the PV may result in increased venous pressure and the formation of ectopic varices around the choledochojejunostomy. Because of this anatomical relationship, patients with PV stenosis are at risk of life-threatening hemorrhage from varices at this site.

PV stenosis after pancreatic or biliary surgery has been attributed to several factors, including postoperative scarring and fibrosis at the site of reconstruction, local tumor recurrence, perivascular inflammation, and technical difficulties during vascular resection and anastomosis [1,2]. These mechanisms can lead to progressive narrowing or occlusion of the PV, ultimately resulting in portal hypertension and the development of ectopic varices.

Previous studies have reported that PV stent placement after pancreatic or biliary surgery is technically feasible in the majority of cases, with technical success rates exceeding 90 % and most patients achieving improvement of portal hypertension-related symptoms [[3], [4], [5]]. Nevertheless, potential complications such as stent restenosis, thrombosis, or rebleeding have been described, and long-term patency remains a clinical concern [6,7]. These findings highlight both the efficacy and limitations of PV stenting, underscoring the importance of further case accumulation.

We describe two patients who underwent successful placement of a PV stent for PV obstruction or stenosis, which led to an improvement in bleeding from varices at a choledochojejunostomy site. This work has been reported in accordance with the SCARE 2025 criteria [8].

## 2 Case presentation

### 2.1 Case 1

A 60-year-old man with stage IB cancer of the pancreatic head was scheduled for surgery after receiving two cycles of neoadjuvant chemotherapy (S-1 and gemcitabine). However, grade 2 eczema and grade 3 neutropenia developed and chemotherapy was discontinued after a single course. He then underwent a subtotal stomach-preserving pancreatoduodenectomy (PD) with resection and reconstruction of the PV, which appeared to have been invaded by tumor. The reconstruction was performed with an end-to-end anastomosis using a continuous 5–0 nonabsorbable suture. Operation time and estimated blood loss were 681 min and 750 mL, respectively. Postoperatively, a thrombus was detected in the left branch of the PV, prompting direct oral anticoagulant administration. Additionally, he experienced delayed gastric emptying after surgery, which required temporary fasting. The patient was eventually discharged home on postoperative day 20.

**Fig. 1:**
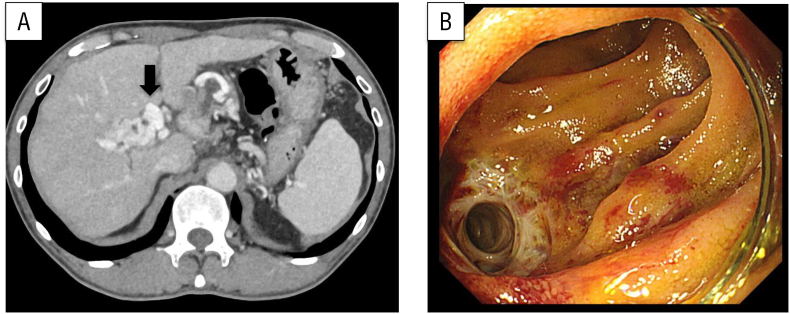
(A) Computed tomography showed ectopic varices around the choledochojejunostomy site (arrow). (B) Enteroscopy revealed ectopic varices around the elevated jejunum at the choledochojejunostomy site.

**Fig. 2:**
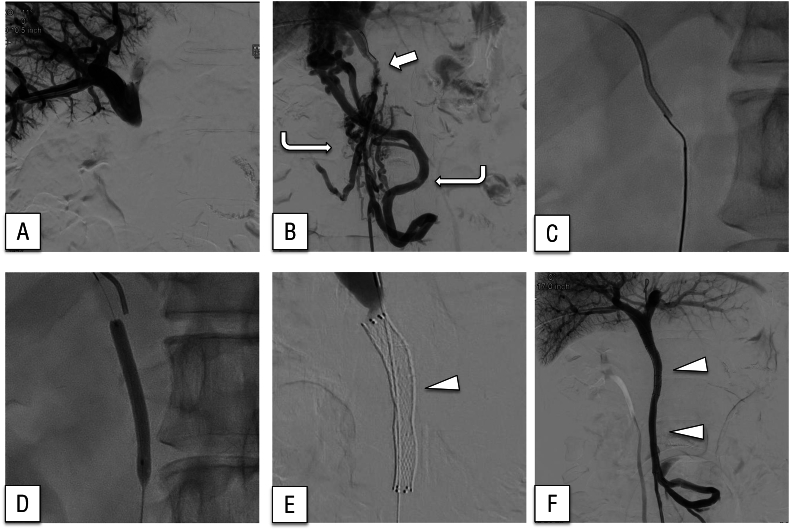
Venography imaging during stent placement. (A) Portography did not show the superior mesenteric vein because of portal vein occlusion. (B) Superior mesenteric venography showing severe narrowing of the portal vein at the anastomotic site (arrow) and the development of tortuous collateral vessels bypassing the occluded segment (curved arrow). (C) Using an antegrade approach, a guidewire was employed to traverse the occlusion. (D) The narrowed area was dilated with a balloon. (E, F) Fluoroscopic images during stent deployment. The expandable-wall stent (**arrowhead**) was successfully placed across the portal vein occlusion, restoring portal venous flow and leading to the disappearance of collateral vessels.

**Fig. 3:**
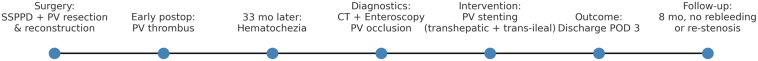
Timeline of Case 1. The patient underwent a subtotal stomach-preserving pancreatoduodenectomy (SSPPD) with portal vein (PV) resection and reconstruction. In the early postoperative period, a PV thrombus was detected. Thirty-three months later, the patient developed hematochezia. Diagnostic work-up with contrast-enhanced computed tomography (CT) and enteroscopy revealed ectopic varices at the choledochojejunostomy site caused by PV occlusion. PV stenting was performed using a combined transhepatic and trans-ileal venous approach. The postoperative course was uneventful, and the patient was discharged on postoperative day 3. At the 8-month follow-up, there was no evidence of rebleeding or PV restenosis.

He later experienced multiple episodes of hematochezia and underwent a comprehensive diagnostic evaluation; however, the underlying cause was not identified. Thirty-three months after surgery, blood testing revealed a hemoglobin concentration of 8.3 g/dL, a considerable decrease from 13.0 g/dL 3 months earlier. Emergency contrast-enhanced computed tomography (Fig. 1A) and enteroscopy revealed ectopic varices around an elevated jejunum at the choledochojejunostomy site (Fig. 1B). Bleeding from the same site was suspected. The patient was urgently admitted and stabilized with a blood transfusion. PV hypertension secondary to PV occlusion was identified as the underlying cause of the varices, prompting consideration of PV stent placement. Percutaneous transhepatic and trans-ileal venous approaches to the occlusion site were performed under general anesthesia. The posterior segmental branch of the PV was percutaneously punctured under ultrasonographic guidance and successfully cannulated (Fig. 2A). Through a lower abdominal midline small incision, the terminal ileum was exteriorized, followed by puncture of the ileocolic vein and cannulation of the superior mesenteric vein (SMV), which exhibited a pressure of 22 mmHg and complete occlusion. Imaging of the obstructed region was performed from both the posterior segmental branch of the PV and the SMV. Collateral circulation directed toward the hepatic hilum was identified (Fig. 2B). Despite encountering technical difficulty, the bidirectional approach enabled application of opposing forces, facilitating guidewire traversal through the occluded segment (Fig. 2C), which was subsequently managed with balloon dilation (Fig. 2D). Two 4 cm bare stents (7 and 8 mm diameter, respectively; Fig. 2E) were deployed within the stenotic segment. Another stenotic lesion was identified caudal to the SMV, where an 8-cm bare stent with a 7 mm diameter was placed following balloon dilation. The collateral circulation was no longer present (Fig. 2F). After stenting, the SMV pressure decreased to 10 mmHg. The procedure time was 166 min. The patient's postoperative recovery was uneventful, and he was discharged home on postoperative day 3. At the 8-month follow-up, no evidence of PV re-occlusion or rebleeding was observed. A timeline of presentation, diagnostic findings, interventions, and outcomes is summarized in Fig. 3.

### 2.2 Case 2

**Fig. 4:**
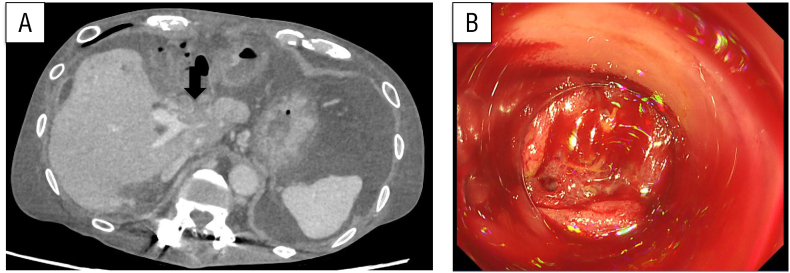
(A) Computed tomography showed portal vein stenosis caused by tumor recurrence (arrow). (B) Enteroscopy revealed bleeding from varices at the choledochojejunostomy site.

**Fig. 5:**
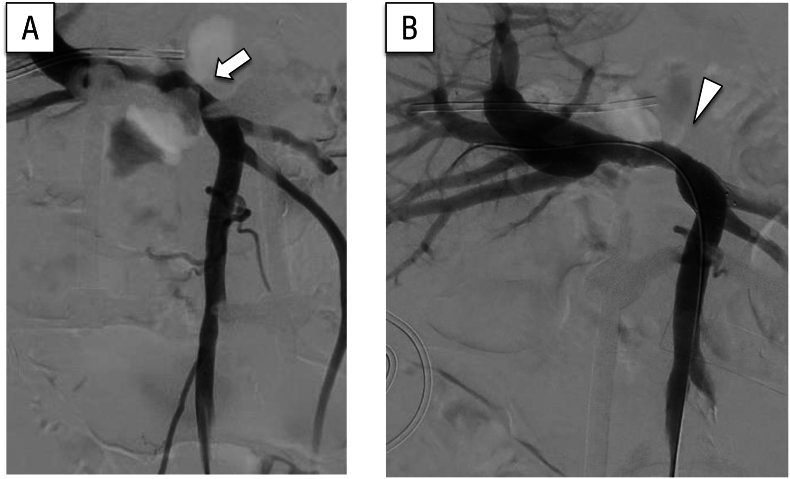
(A) Portal vein stenosis was identified via contrast imaging conducted through the ileocolic vein (arrow). (B) A stent was deployed at the site of portal vein stenosis, resulting in observable improvement of the narrowing (arrowhead).

**Fig. 6:**
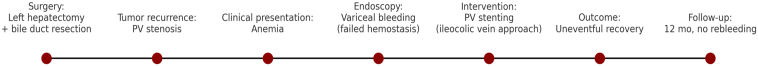
Timeline of Case 2. The patient had a history of left hepatectomy with extrahepatic bile duct resection and reconstruction for hilar cholangiocarcinoma. He subsequently developed tumor recurrence associated with portal vein (PV) stenosis. During follow-up, the patient presented with anemia. Endoscopic evaluation revealed variceal bleeding at the choledochojejunostomy site, but endoscopic hemostasis was unsuccessful. PV stenting was then performed via the ileocolic vein approach. The postoperative course was uneventful, and at the 12-month follow-up there was no recurrence of gastrointestinal bleeding.

An 82-year-old man with a history of left hepatic lobectomy and extrahepatic bile duct resection and reconstruction for stage III hilar cholangiocarcinoma presented with tumor recurrence in the hilar region. Adjuvant chemotherapy had not been administered at the patient's request. Although pharmacological treatment had been planned, he developed a liver abscess, which was managed with percutaneous transhepatic drainage. While recovering, his anemia worsened and he reported melena. Fecal occult blood testing was positive. Lower gastrointestinal endoscopy failed to identify the bleeding source; however, enteroscopy revealed hemorrhage from varices at the site of the bile duct–jejunostomy. Endoscopic hemostasis was performed, followed by repeat endoscopy 5 days later, during which rebleeding was observed. Fecal occult blood testing remained positive and he required multiple blood transfusions. Further evaluation identified PV hypertension and stenosis secondary to the recurrent tumor (Fig. 4A.B). A PV stent was then placed via the ileocolic vein under general anesthesia. Access was obtained through the ileocolic vein, revealing stenosis within the PV (Fig. 5A). Prior to stent deployment, pressure measurements in the SMV and PV were 27 and 12 mmHg, respectively. A 4 cm bare stent with 12 mm diameter was positioned to cover the stenotic region from the anterior-posterior segment to the confluence of the splenic vein. Balloon dilation was also performed (Fig. 5B). Following stent placement, the SMV and PV pressures were 20 and 17 mmHg, respectively. At the 12-month follow-up, no recurrence of gastrointestinal bleeding was identified. A timeline of presentation, diagnostic findings, interventions, and outcomes is summarized in Fig. 6.

## 3 Discussion

As illustrated by the two cases presented here, PV stenting can be used to manage variceal bleeding at a choledochojejunostomy site resulting from PV obstruction. Moreover, it can be safely performed using both percutaneous transhepatic and trans-ileal venous approaches.

The estimated incidence of PV stenosis following PD is 19.6 %, and approximately half of post-PD PV complications are attributed to stenosis after resection and reconstruction [2]. PV stenosis results in PV hypertension, which may then give rise to varices. In patients who have undergone PD, these varices commonly develop at the choledochojejunostomy site [9]. Bleeding from varices has been reported in approximately 3 % of PV stenosis cases [2]. In case 1, PV obstruction developed in a patient who had undergone PD with PV reconstruction, and variceal bleeding at the choledochojejunostomy site developed. Despite a thorough evaluation, identifying the bleeding source required considerable time. In such patients, clinicians should consider the possibility of ectopic variceal bleeding at or near the choledochojejunostomy site, and pursue an in-depth diagnostic assessment accordingly.

When managing variceal bleeding at a choledochojejunostomy site caused by PV stenosis, it is crucial to consider two therapeutic approaches: one targeting the ectopic varices (re-anastomosis, endoscopic sclerotherapy, and variceal embolization) and the other addressing the PV stenosis (endovascular or surgical portosystemic shunt and PV stenting) [10,11]. Although successful outcomes have been reported with embolization of ectopic varices alone [12], the risk of recurrence is high if the treatment fails to resolve the PV hypertension caused by extrahepatic PV stenosis or obstruction [13]. In case 1, the PV stenosis, the underlying cause of PV hypertension, was directly addressed. Surgical creation of a shunt was deemed high-risk because of adhesions from his prior surgery and the potential for intraoperative bleeding from collateral vessels; therefore, PV stenting was selected as the treatment approach. Embolization may provide temporary hemostasis but does not resolve the underlying portal hypertension and carries a risk of rebleeding. Surgical shunts can achieve more definitive decompression, but they are highly invasive and particularly risky in patients who have undergone major hepato-biliary-pancreatic surgery such as PD. Dense adhesions and fragile collateral vessels are frequently present in these patients, and injury to these vessels during adhesiolysis may result in uncontrollable hemorrhage. Thus, surgical shunts are often not feasible in this setting. In contrast, PV stenting directly restores portal venous flow at the site of obstruction, reducing portal pressure in a less invasive and safer manner.

We have reviewed previously reported cases in which PV stent placement was used to treat variceal bleeding at the choledochojejunostomy site caused by PV stenosis or occlusion after PD by conducting a literature search in PubMed using the keywords “pancreatoduodenectomy”, “portal vein stent placement” and “gastrointestinal bleeding”. Twenty-two patients have been reported to date (Table 1) [1,[3], [4], [5],^7^,^9^,^14^,^15^]. Only one case was reported for PV occlusion, and 20 cases were reported for portal vein stenosis with portal vein stent placement. PV stent placement was successful in 21 of the 22 cases. Post-procedural administration of an oral antiplatelet agent was reported in 14. In two, embolization of collateral vessels was performed concurrently. Hemorrhage from ectopic varices resolved in all patients who underwent successful PV stenting. Stent occlusion occurred in only one case, 46 months after the procedure. Bleeding improved in all reported cases, suggesting that PV stent placement may be a viable option for treating variceal bleeding at a choledochojejunostomy site caused by PV stenosis or occlusion.

**Table 1:** Cases of portal vein stenting after pancreatoduodenectomy.

No.	First author	Year	n	Postoperative period (months)	Patency period (months)	approach	Technical success	Antithrombotic therapy	Embolization of collateral veins	Improvement of symptoms	Stent occlusion
1	Odaira [2]	2024	1	18	21	PTP	1/1	yes	yes	1/1	0/1
2	Megu [7]	2020	6	NS	NS	PTP	6/6	yes	no	6/6	NS
3	Ohgi [8]	2019	6	8–95	14–104	PTP	6/6	NS	no	6/6	NS
4	Kasper [9]	2019	1	48	NS	PTP	1/1	NS	no	1/1	0/1
5	Hiyoshi [10]	2015	5	NS	21–41	PTP	4/5	yes	no	4/4	1/4
6	Sakurai [11]	2014	1	24	7	TIC	1/1	yes	yes	1/1	0/1
7	Ellis [12]	2009	1	15	4	PTP	1/1	NS	no	1/1	0/1
8	Ota [13]	2005	1	98	32	PTP	1/1	yes	no	1/1	0/1

NS, not stated; PTP, percutaneous transhepatic portal vein approach; TIC, transileocolic vein approach.

PV stent placement can be performed via two primary approaches: the percutaneous transhepatic approach and the trans-ileal venous approach. As shown in Table 1, the percutaneous transhepatic approach was used in most of the previously reported cases. This approach is generally preferred because of its less invasive nature. Except for one case, all instances in Table 1 involved PV stenosis. In such cases, the PV pathway can typically be visualized using a contrast agent, allowing for safe stent placement via the percutaneous transhepatic approach. However, in cases of complete PV obstruction, blindly advancing a guidewire poses a risk of vascular injury. To mitigate this risk and enable thorough assessment of the obstructed segment, we employed a rendezvous technique combining both the percutaneous transhepatic and trans-ileal venous approaches to facilitate safe and accurate guidewire navigation. In case 2, although percutaneous transluminal angioplasty was considered feasible, the procedure was conducted via the ileocolic vein approach because of our familiarity with this route, which we commonly use for portal vein embolization. Although the combined transhepatic and trans-ileal venous approach is not commonly reported, its use in our case was intended to ensure secure access in the setting of portal vein occlusion. This dual-route strategy facilitated safe guidewire manipulation and stent deployment without any perioperative complications or technical difficulty. In contrast, in cases of portal vein stenosis rather than occlusion, a single familiar approach is generally sufficient for safe stent placement. These considerations suggest that the optimal access strategy should be tailored to the underlying pathology: bilateral approaches for complete occlusion, and unilateral approaches for stenosis.

The use of antiplatelet and anticoagulant therapy after PV stent placement remains a subject of ongoing debate. While such therapy is generally considered necessary to prevent stent occlusion, Chen et al. reported no significant correlation between antithrombotic therapy and PV stent patency [6]. To date, reports of PV stenting for hemorrhage from ectopic varices secondary to PV stenosis after PD remain scarce, and no study has specifically evaluated whether post-stenting antithrombotic therapy increases the risk of variceal rebleeding. Comparative analysis was not feasible based on the cases in Table 1, owing to lack of sufficient data reporting. Further case accumulation and investigation are warranted to clarify this issue. In our cases, antithrombotic management was individualized according to clinical context. In Case 1, anticoagulation with warfarin was initiated after PV stenting and maintained during follow-up. In Case 2, no antithrombotic therapy was given because of the concern for rebleeding. These cases highlight that antithrombotic therapy after PV stenting should be tailored to the balance between stent patency and bleeding risk, as no consensus exists in this specific clinical scenario.

Both patients reported relief from the anxiety and distress associated with recurrent bleeding after portal vein stenting. They expressed satisfaction with the improvement in their quality of life and the absence of further bleeding episodes during follow-up.

## 4 Conclusions

In conclusion, these cases demonstrate that portal vein stenosis or occlusion should be considered in the differential diagnosis of variceal bleeding at the choledochojejunostomy site following hepato-biliary-pancreatic surgery. Careful diagnostic evaluation is crucial for timely recognition of this rare but potentially life-threatening condition. Furthermore, portal vein stenting can serve as a safe and effective treatment option, particularly when endoscopic hemostasis is unsuccessful. These clinical lessons highlight the importance of early suspicion, multidisciplinary management, and the potential role of PV stenting in preventing recurrent bleeding in selected patients.

## Abbreviations

PD pancreatoduodenectomy

PV portal vein

SMV superior mesenteric vein

## Ethical approval

Ethics approval and consent for publication of this case report and any accompanying images were obtained.

## Funding

No funding was received specifically for this work.

## Author contribution

N.I. participated in the writing of the manuscript. R.M., K.K., T.Y., and H.O. participated in data collection. R.M., K.K. T.Y. and T.N., reviewed the article. All authors read and approved the final manuscript.

## Conflict of interest statement

The authors declare that they have no known competing financial interests or personal relationships that could have appeared to influence the work reported in this paper.

## Guarantor

Norifumi Iseda.

## Research registration number

Not applicable

## Consent

Written informed consent was obtained from the patients for publication of this case report and any accompanying images.

## Acknowledgments

We thank Edanz (https://jp.edanz.com/ac) for editing a draft of this manuscript.

## Data Availability

Not applicable.

## References

[1] Ohgi K., Sugiura T., Yamamoto Y., Okamura Y., Ito T., Ashida R. (2019). Benign portal vein stenosis after Pancreaticoduodenectomy. World J. Surg..

[2] Kang M.J., Jang J.Y., Chang Y.R., Jung W., Kim S.W. (2015). Portal vein patency after pancreatoduodenectomy for periampullary cancer. Br. J. Surg..

[3] Ota S., Suzuki S., Mitsuoka H., Unno N., Inagawa S., Takehara Y. (2005). Effect of a portal venous stent for gastrointestinal hemorrhage from jejunal varices caused by portal hypertension after pancreatoduodenectomy. J. Hepato-Biliary-Pancreat. Surg..

[4] Mugu V.K., Thompson S.M., Fleming C.J., Yohanathan L., Truty M.J., Kendrick M.L. (2020). Evaluation of technical success, efficacy, and safety of portomesenteric venous intervention following nontransplant hepatobiliary or pancreatic surgery. J Vasc Interv Radiol JVIR.

[5] Hiyoshi M., Fujii Y., Kondo K., Imamura N., Nagano M., Ohuchida J. (2015). Stent placement for portal vein stenosis after Pancreaticoduodenectomy. World J. Surg..

[6] Chen L., Wang Z., Dong L., Wang Z., Li Z., Wang W. (2024). Comparison of patency rates and complications with or without antithrombotic therapy following portal vein stent placement after pancreatic surgery: a systematic review and meta-analysis. Int J Surg Lond Engl..

[7] Kasper P., Schramm C., Jaspers N., Goeser T. (2019). Jejunal varices as a rare cause of recurrent gastrointestinal bleeding in a 74-year-old man with extrahepatic portal hypertension after pancreato-biliary surgery. BMJ Case Rep..

[8] Kerwan A., Al-Jabir A., Mathew G., Sohrabi C., Rashid R., Franchi T. (2025). Revised surgical CAse REport (SCARE) guideline: an update for the age of artificial intelligence. Premier Journal of Science.

[9] Odaira M., Ito N., Iwaita Y., Tanuma K., Harada H. (2024). Percutaneous Transhepatic venous embolization and portal vein stenting for ectopic variceal bleeding at Choledochojejunostomy after Pancreaticoduodenectomy with portal vein stenosis: a case report. Cureus.

[10] Li M., Li Q., Lei Q., Hu J., Wang F., Chen H. (2018). Unusual bleeding from hepaticojejunostomy controlled by side-to-side splenorenal shunt: a case report. Medicine (Baltimore).

[11] Saeki Y., Ide K., Kakizawa H., Ishikawa M., Tashiro H., Ohdan H. (2013). Controlling the bleeding of jejunal varices formed at the site of choledochojejunostomy: report of 2 cases and a review of the literature. Surg. Today.

[12] Sasamoto A., Kamiya J., Nimura Y., Nagino M. (2010). Successful embolization therapy for bleeding from jejunal varices after choledochojejunostomy: report of a case. Surg. Today.

[13] Norton I.D., Andrews J.C., Kamath P.S. (1998). Management of ectopic varices. Hepatology.

[14] Sakurai K., Amano R., Yamamoto A., Nishida N., Matsutani S., Hirata K. (2014). Portal vein stenting to treat portal vein stenosis in a patient with malignant tumor and gastrointestinal bleeding. Int. Surg..

[15] Ellis C.M., Shenoy S., Litwin A., Soehnlein S., Gibbs J.F. (2009). Effective endovascular stenting of malignant portal vein obstruction in pancreatic cancer. HPB Surg World J Hepatic Pancreat Biliary Surg..

